# Supplementary material for: Are carbon emissions trading and green financial instruments synergistic? -Comprehensive quantitative research based on content analysis
Source: PLoS One. 2024 Mar 7;19(3):e0298601. doi: 10.1371/journal.pone.0298601 (PMC10919601; doi:10.1371/journal.pone.0298601)
Supplement: S1 Data — (ZIP) [file pone.0298601.s001.zip › Data/地级市Z_F工作报告.zip!/╣π╢1⁄2/╜¡├┼╩╨/└┤╫╘╣1⁄2╓┌║┼í╛┬φ┐╦╩2╛▌═°í┐_2006.docx]

各位代表：

　　我代表市人民政府向大会作政府工作报告，请予审议，并请市政协委员和各位列席人员提出意见。

　　“十五”时期的工作回顾

　　“十五”时期，江门侨乡经济和社会发展上了一个新台阶。全市上下团结一心，抢抓机遇，开拓进取，战胜非典型肺炎和百年一遇特大洪灾，呈现经济持续健康发展，各项事业全面进步，社会发展繁荣稳定的良好局面，圆满完成市十二届人大四次会议确定的２００５年各项预期目标，基本实现市十一届人大三次会议通过的“十五”计划各项任务。

　　（一）经济步入加快发展轨道。２００５年，全市生产总值８０２．１６亿元，比上年增长１２．７％（按可比口径计算，下同），高于全省平均增速，创近１０年新高；“十五”期间年递增１０．５８％，比“九五”时期提高１．４４个百分点。地方财政一般预算收入４１．６３亿元，比上年增长１９．６９％，“十五”时期年递增１６．９１％，近三年超过全省财政收入平均增速；地方财政一般预算收入占ＧＤＰ比重由“九五”期末的４．２％升至５．２％。全市城乡居民储蓄存款余额８５１．９３亿元，城镇在岗职工人均年工资收入１５０３０元，农村居民人均年纯收入５３６６元，比“九五”期末分别增长７６％、５７．８％和２１．１％。

　　临港经济崛起。正在形成以银洲湖和广海湾为重点区域的新增长极。电力、造纸、钢铁、建材、物流等临港产业渐具雏型。台山电厂４台机组和新会双水电厂２台机组建成运营，新增装机容量２７０万千瓦，台山核电站、新会电厂前期建设展开；海螺水泥、华冠钢铁、华尔润玻璃等大型项目拔地而起，全省第二大利用外资项目亚太纸业落户银洲湖畔。

　　产业集聚能力增强。２００５年，全市规模以上工业总产值和增加值分别达１４４３．２８亿元和３１４．７８亿元，比上年增长１７．７３％和１６．４４％，“十五”时期年递增１５．４２％和１４．４３％。建成摩托车、电子信息、五金卫浴、纺织化纤、不锈钢制品、纺织、水龙头、新材料、麦克风、纳米碳酸钙１０个国家级产业基地，形成一批较为完整的产业链，打造出“豪爵”摩托车等９个中国名牌产品，数量居全省第４位。“三大组团”快速发展，市高新区、江沙工业走廊和银洲湖区域规模以上工业总产值分别达６８亿元、１６３亿元和２９４亿元，其中，银洲湖区域引进项目逾４００个，总投资超４００亿元。

　　（二）结构调整优化迈出重大步伐。“十五”期间，三次产业结构由１３．８：４６．６：３９．６演进为９．９∶４９．６：４０．５。产业高级化和重型化趋势明显，重工业所占比重由“九五”期末的３３％提升至４２％。高新技术产业发展迅速，高新技术企业达１５８家，比“九五”期末增加８５家；高新技术产品产值占工业总产值比重由“九五”期末的１３．１％提高到２５．５％。信息化建设成效显著，全市信息化综合指数达７１．５１％，比“九五”期末提高３０．８３个百分点。

　　外向型经济上新水平。２００５年，全市进出口总额达９０．５亿美元，其中出口总额６０．２亿美元，比上年增长１８．６％，“十五”时期年均增长１５．０８％。机电和高新技术产品占出口总额的比重，分别由“九五”期末的４５．４６％和６．３８％提高至５６．７８％和８．０１％。２００５年实际吸收外商直接投资６．０６亿美元，比上年增长１８．３９％；“十五”时期累计实际吸收外商直接投资３５．２９亿美元，世界５００强企业有２２家落户我市。成功举办世界银行中国投资环境论坛、粤台经贸交流会和机电博览会。

　　民营经济快速成长。２００５年，全市规模以上民营企业实现工业总产值５０８．５９亿元，比上年增长１８．４１％，占全市工业总产值３５．５％，比“九五”期末提高１０．７个百分点。私营企业户数和注册资金分别达１．４３万户和１７１．１亿元，比“九五”期末增长７２％和２．５倍。

　　服务业发展创新局面。２００５年，第三产业增加值３２４．５８亿元，比上年增长１１．７％，“十五”时期年均增长１０．５２％；社会消费品零售总额３０９．８２亿元，比上年增长１２．３％，“十五”时期年均增长１１．８％。物流业、旅游业等新兴服务业发展势头良好，江门大昌慎昌公用型保税仓挂牌运作，兴建了逸豪、丽宫国际等五星级酒店，新增温泉、碉楼、崖门宋元古战场等旅游文化景区，２００５年共接待过夜游客５７５．２５万人次，旅游总收入４９．７亿元，比上年分别增长６．４％和８．０％。

　　（三）基础设施和城乡建设获重大进展。２００５年，全市固定资产投资２３７．４６亿元，比上年增长１８．６４％，江中高速和江鹤高速二期、３２５国道开平段改造工程、江会路复线、江海路改造和东海路扩建工程建成通车，滨江大道工程完成投资逾三分之一。“十五”期间，累计完成固定资产投资８５６亿元，比“九五”时期增长８９％；全市公路通车里程６８２８公里，每百平方公里公路密度为７１．６公里；高速公路通车里程新增２６３公里，总长度３３０公里，居全省地级市首位，全国地级市第二位；农村公路硬底化建设惠及群众６０多万人。新会港被国家列为一类货运口岸，西江下游航道和潭江航道整治工程全面完成，劳龙虎水道和崖门出海航道整治工程开工，广海湾及川岛深水港规划研究工作取得阶段性成果。

　　城镇化进程加快，按户籍口径计算，全市城镇化水平由“九五”期末的３５％提高到５２％。共撤并２６个镇，撤并率为２８％，新会撤市设区，扩大了中心城区发展空间。完成市区供水并网，实施江门河综合整治，建成３家污水处理厂。市区生活垃圾无害化和污水处理率分别达１００％和６３．４％，空气日报质量优良率达９７．０％，人均公共绿地面积９．５２平方米。荣获“国家园林城市”、“国家卫生城市”、“国家环境保护模范城市”和“中国人居环境范例奖”。

　　（四）“三化”促“三农”取得新成效。出台了鼓励边远困难地区异地发展工业的政策，镇级经济实现新突破。２００５年全市镇级地方财政一般预算收入１６．５２亿元，比上年增长２２．９１％；财政收入超千万元镇达４５个，比“九五”期末增加２３个。全力防治高致病性禽流感，全市未发生重大动物疫情。农业结构调整取得新成效，粮经作物面积比由“九五”期末的６１：３９调整为５５：４５，全市农作物良种覆盖率保持９５％以上。农业产业化进程加快，全市农业产业化组织发展到２７８个，其中农民专业合作经济组织１２８个，农业龙头企业１５０家，带动农户近２０万户。农村生产生活条件明显改善，取消农业税，降低农村电价，农民人均税费负担比“九五”期末减少９９％。完成８８４项水利达标加固工程，实现了１９９８年市人大议案提出的目标。全市农村自来水和液化气普及率分别达到８１％和９０％，解决了３７０条村２０．７万人的饮水难问题。扶贫力度加大，２６４条年集体收入３万元以下的贫困村实现脱贫。

　　（五）体制改革实现新突破。“十五”期间，市直国（公）有企业累计实施改制３９６户，市政府授权经营的２１２户国有企业改制面从“九五”期末的６．６％扩至９５％，累计安置市直职工３．８万人，发放职工安置补偿费近６亿元。建立健全三级国有资产管理体制，成立了国有资产监督管理委员会和产权交易中心。甘化股份在全省首创“以股抵债”的股改模式，恩平广联泰、江门市食品工业集团实施破产等重大资产处置和债务重组工作取得显著成效。机构改革、行政审批制度改革和公共财政体制改革取得重要进展，投资体制改革稳步推进，农信社体制改革圆满完成，增资扩股超额完成省下达的目标。

　　（六）社会各项事业协调发展。群众性精神文明创建活动深入开展，被评为省文明城市。文化名市建设扎实推进，建成五邑华侨广场、五邑华侨华人博物馆、广东桥梁博物馆、江门美术馆等文化馆场及长堤风貌街、院士路等标志性景观。成功举办首届华人嘉年华、中国曲艺节、广东国际旅游文化节、侨乡旅游节和书香节等一系列活动。

　　教育事业均衡发展。扩大高等教育办学规模，适龄青年高等教育毛入学率由“九五”期末的１４．９％提高到２７．１９％，五邑大学办学质量逐年提高，新组建江门职业技术学院。高中阶段教育毛入学率提高了２０．１个百分点，达７７．６％，建成江门一中新校区，国家级重点中等学校数和在校生人数居全省第二位。职业教育蓬勃发展。完成４０５所中小学布局调整和８１所老区、山区小学改造，创建省一级学校６７所，市一级学校１３７所。人才培养工作不断强化，截至２００５年底，全市人才资源总量达２９．２２万人，比“九五”期末增长３３．０６％。

　　社保和再就业工作力度加大。“十五”期间，全市新增城镇就业岗位１５．２万个，安置城镇人员就业近１０万人，下岗失业人员实现再就业２．９万人，“４０５０”人员实现再就业１万人，城镇登记失业率控制在３％以下。社会保障体系进一步完善，２００５年全市参加养老、失业、医疗和工伤保险人数分别达４７．１８万、３８．３９万、４４．８８万和３３．６２万人，比“九五”期末分别增长２４％、５．４％、２２％和２２％。

　　公共卫生防控体系及服务网络进一步完善。建成市疾控中心，台山市成为全省首批艾滋病综合防治示范区。新型农村合作医疗加快推进，全市参加农村合作医疗人口１６９．１７万人，覆盖率达７１．２７％。

　　其他社会事业取得新成绩。计划生育工作巩固提高，全面完成省下达的人口计划任务。社会福利事业日益蓬勃，开展慈善公益万人行活动。大力推进全民健身活动，创建体育先进社区工作初见成效，市体育公园投入使用，“国家网球训练基地”落户我市，江门五邑籍运动员在国内外重大赛事中取得优异成绩。人防建设上新水平，国防动员、民兵预备役和双拥优抚安置工作进一步加强，荣获“全国双拥模范城”三连冠。外事、侨务、港澳台事务工作有新进展。新闻、出版、档案、地志、宗教、老龄、气象等各项工作有新进步。

　　社会大局保持稳定。严厉打击各种违法犯罪活动，禁毒０３工程取得明显成效，社会治安进一步好转。贯彻落实《信访条例》，正确处理人民内部矛盾。“三防”、安全生产、食品安全、消防安全、道路交通安全等关系群众和社会安全的基础工作进一步夯实。

　　（七）政府行政能力显著提高。自觉接受市人大及其常委会和市政协的监督，认真执行市人大及其常委会的决议，多途径听取人大代表和政协委员的意见建议。进一步完善办理人大建议、政协提案工作网络，“十五”期间共办理人大代表议案２件；办理人大代表建议７０２件，办理结果满意率为９６．４％；办理政协提案７３６件，办理结果满意率为９６．６％。认真实施行政许可法，依法行政水平进一步提高。加强民本政府建设，大力推进“十项民心工程”，在全省率先开通“江门市政府网上行政服务系统”，开设网上“侨乡论坛”，推行“群众点题”等政务公开做法，设立“１２３４５”政府为市民服务热线。建立全市经济形势分析联席会议和经济社会发展顾问咨询制度，健全重大决策和重要规划的论证和听证程序，完成《江门市可持续发展战略纲要》和《江门市国民经济和社会发展第十一个五年规划纲要（草案）》的起草工作，委托五邑大学开发城市经济发展预测与决策支持系统软件。简化审批程序，清理审批事项。在市直机关全面推行ＩＳＯ质量管理体系，实施推诿、搁置追究制，健全投诉机制，政府工作的规范性和效能进一步提高。深入开展纠正损害群众利益不正之风的专项治理工作，查处了一批违纪违法案件，政府廉政建设和反腐败工作扎实推进。

　　各位代表，经过“十五”时期的努力，我市经济社会发展开创了新的局面。这是贯彻落实党的十六大精神、“三个代表”重要思想和科学发展观的结果，是全市人民和中央、省驻我市单位及驻军警部队团结奋斗的结果，是各民主党派、社会各界人士、外来投资者和建设者、港澳台同胞、海外侨胞关心支持的结果。在此，我代表市人民政府，向参与和关心支持江门经济社会发展的同志们、朋友们、同胞们表示崇高的敬意和衷心的感谢！

　　在发展的同时，我市经济社会发展还存在不少不容忽视的问题，主要是：经济发展仍处于依靠资源投入为主的数量扩张型阶段，自主创新能力弱；城乡区域发展不平衡问题依然突出，农民收入增长较慢；影响社会稳定的因素增多，维稳工作难度加大；医疗卫生、城市管理、社会治安、政府自身建设等方面还存在薄弱环节。对这些问题，市政府正采取切实有效措施加以解决。

　　“十一五”时期的奋斗目标和２００６年的主要工作

　　“十一五”时期是我市落实科学发展观、实现后发争先，建设全面协调可持续发展的现代化大城市的关键时期。我们处在总体上有利的发展环境中，国际国内形势给我们提供了难得的发展机遇，也使我们面临更多的挑战和困难。只要我们在“十五”发展的坚实基础上，坚持科学发展观不动摇，全市上下一心，开拓创新，扎实奋斗，就一定能在新的起点 上，全面开创我市“十一五”时期经济社会发展的新局面。

　　根据中共江门市委十届五次全会精神，“十一五”时期我市经济社会发展的指导思想是：以邓小平理论和“三个代表”重要思想为指导，以科学发展观统领经济社会发展全局，深入实施工业强市、民资外资富市、科教兴市、环境优市发展战略，优化经济结构，加强自主创新，实现增长方式的根本性转变，统筹城乡和社会发展，建设富裕、绿色、和谐江门，为基本实现社会主义现代化迈出更大的步伐。

　　“十一五”时期我市经济社会发展要实现以下预期目标：

　　--在增加经济发展总量方面，在优化结构、提高效益和降低消耗的基础上，保持经济持续快速健康发展，全市生产总值年均增长１１％以上，到２０１０年实现ＧＤＰ超１３００亿元，人均ＧＤＰ比２０００年翻一番以上。

　　--在优化结构、转变经济增长方式方面，资源利用效率进一步提高，单位生产总值能源消耗比“十五”期末降低１３％以上；建成广东省重要的能源基地和现代化交通运输体系，物流和金融服务体系建设取得重大进展，电子信息、汽车零配件、精细化工、精密制造等先进制造业和临港重化工业加快发展，经济结构更趋合理，到２０１０年三次产业比例达到６：５３：４１，高新技术产业增加值占工业增加值的比重达到３５％以上。

　　--在提高自主创新能力方面，基本建立以企业为主体、市场为导向、产学研相结合的区域自主创新体系和比较完善的公共服务平台。到２０１０年全市财政科技经费投入占财政支出的比重达到２％以上，全市科技进步贡献率和技术自给率分别达到５３％和４８％，省、市级工程技术研究开发中心达到１５０家以上，创中国名牌产品保持全省前列，创新型江门建设取得重大突破。

　　--在促进内外源经济协调发展方面，建成比较完善的社会主义市场经济体制，开放型经济再上新水平，形成外源经济和内源经济相互促进、协调发展的新格局。市高新区、银洲湖开发区、江沙工业走廊和广海湾开发区等核心工业园区的建设有较大发展。民营经济发展环境全面优化，到２０１０年民营工业占全市工业比重达到４０％以上。引进外资的规模和质量有较大提高，“十一五”期间实际吸收外商直接投资４０亿美元以上，出口商品结构进一步优化，２０１０年机电产品和高新技术产品的出口比重分别达到６５％和１５％以上。

　　--在建设社会主义新农村方面，按照“生产发展、生活宽裕、乡风文明、村容整洁、管理民主”的要求，建立以工促农、以城带乡的长效机制。边远后进地区与富裕地区、城市与乡镇协调发展，中心镇的城镇化和工业化水平明显提高，２０１０年全市城镇化水平达到６５％，非农就业比重达到３５％。农业龙头企业和农民专业合作经济组织有较大发展，农业综合效益显著提高，农民收入增幅接近城市居民。农村教育、卫生和文化等事业加快发展，全面实行免费义务教育，建立起比较完善的公共服务体系。

　　--在建设绿色江门方面，以节能、节地、节材、节水为重点的经济与资源协同发展战略加速推进，节约型社会建设取得显著成果，生态市建设工作稳步推进。基本建成企业、区域和社会三个层面的循环经济框架。环保监管水平再上新台阶，污染防治与生态保护长效机制初步建立，城市生活污水处理率达到７０％。森林、湿地和野生动物资源得到保护，海岸生态系统优良，人均公共绿地面积达到１２平方米，创建全国绿化模范城市，成为珠三角人居环境最好的地区之一。

　　--在建设文化名市方面，进一步提高公民素质，创建国家文明城市。加快发展文化产业，建立现代公共文化服务体系，繁荣哲学社会科学，进一步弘扬侨乡文化，广泛开展社区文化建设。建成教育强市，２０１０年高等教育毛入学率达到３５％，高中阶段教育毛入学率达到８５％以上，２５岁以上人口平均受教育年限达到１０年以上，幼儿教育、基础教育、职业教育、高等教育和成人教育水平有较大提高。

　　--在建设和谐江门方面，政府的社会管理和公共服务职能得到强化，市民满意度较高，学习型、创新型、服务型、效率型、法治型政府建设上新台阶。就业形势稳定，城镇登记失业率控制在３．８％以内。全市基本建立起城乡社区卫生服务体系。公共安全预警和应急体系健全，社会治安防控体系不断完善，无毒社区和平安社区建设上新水平，社会治安和安全生产保持平稳。社会保障体系逐步覆盖全社会，人民生活总体达到宽裕型小康水平，城镇居民人均可支配收入年均增长７％，到２０１０年达到１８０００元以上；农村居民人均纯收入年均增长５％，到２０１０年达到６８００元以上。

　　今年是实施“十一五”规划的第一年，是夯实基础、开创新局面的关键一年。今年我市经济社会发展的主要预期目标是：生产总值增长１１．５％，单位生产总值能耗比上年下降３％，地方财政一般预算收入增长１２％，全社会固定资产投资增长１５％，外贸进出口增长１０％以上，社会消费品零售总额增长１２％，城镇居民人均可支配收入增长７％，农村居民人均纯收入增长４．５％左右。今年政府工作要重点抓好以下几个方面：

　　一、着力转变经济增长方式，促进产业结构优化升级

　　加快临港经济发展，推动产业布局由沿江向沿海提升。全力以赴抓好川岛深水港规划建设的前期工作，力争列入国家和省的“十一五”港口建设规划。抓好台山直购电试点方案的报批和实施工作，加强对广海湾开发区的领导，加快开发建设步伐。抓住崖门口区域被列入省石化基地的重大机遇，积极发展石化项目和精细化工产业。

　　抓好重大项目建设，推动产业结构由偏轻向适度重型化提升。加快台山电厂一期工程５号机组、台山核电站、新会电厂及川岛风电场前期工程建设，全力争取台山电厂二期、新会古兜抽水蓄能电站立项，加快输变电工程建设，壮大能源基地规模。协调加快大长江集团三期、亚太纸业一期、华冠钢铁、中技沥青、开平纺织城、海螺水泥和维达纸业扩产等大项目建设，做大重化工业规模。

　　提高产业集群发展水平，推动产业环境由“政策洼地”向“产业洼地”提升。大力推进信息化，建立企业基础信息数据交换中心，创建国家信息化示范城市。加快大企业集团和中小企业集群发展，制订摩托车、汽车零配件、石油化工、纺织化纤、电子信息、玻璃建材、印刷、钻石珠宝等产业发展规划，打造更多的国家级和省级制造业“航母”，高标准建设好江门摩托车产业集群升级示范区。

　　二、着力增强自主创新能力，建设创新型江门

　　加快产业公共服务平台建设。把产业扶持政策和财政专项资金向打造产业服务平台倾斜，重点推进摩托车、麦克风、不锈钢、五金卫浴、纺织服装等行业的技术创新服务、质量技术标准和检测检验服务、信息服务、融资服务和专业市场展销等服务平台建设。积极争取上级支持，落实更多技术进步扶持项目。

　　发挥大中型企业、高新技术企业和高校自主创新主力军作用。抓好市高新区申报国家级高新区工作。鼓励企业研发机构创造条件成为省级工程技术研发中心。鼓励企业对关键或重大技术问题开展产学研相结合的科技攻关，重奖作出突出贡献的科技人员。鼓励以行业商会（协会）牵头，建立中小企业技术创新联合体。技术改造要降低审核门槛，简化审核程序，充分发挥财政资金的杠杆作用，调动企业加大技改投入的积极性。

　　大力实施名牌带动战略，推进质量兴市工作。培育发展自主知识产权和品牌，推动产业基地打造区域品牌，力争再创多个“中国名牌产品”及“中国驰名商标”。加强名牌产品、商标等知识产权的保护，为企业营造良好的创新环境。推广国际标准和国外先进标准，鼓励优势企业积极参与行业、国家和国际标准的制订。

　　三、着力促进内外源经济协调发展，培育新经济增长点

　　创新产业招商思路。拓宽以侨引商、以商引商、中介引商等渠道，找准产业转移结合点，既瞄准世界５００强和跨国大集团，又重视引进技术含量高、投资强度大、产业链长的外资民资项目。在扩大引资规模的同时，着力提升引资质量，突出抓好对欧美、日韩企业的招商，强化“亩产”和环保意识，提高土地集约使用率。进一步落实民营经济政策，凡是法律法规未禁止进入的领域都要向民营企业开绿灯。进一步强化对招商引资的服务，建立重大项目主要领导协调督办制度，全面落实首问负责制、全程代办制、限时办结制和服务承诺制。抓好招商队伍建设，扩大招商引资网络。兑现引资奖励政策，调动招商积极性。发挥和扩大摩托车博览会、新会五金不锈钢展览会、水口水暖卫浴展会、开平牛仔服装节、恩平电声器材博览会等会展效应。鼓励外资民资企业增资扩产、设立研发机构和地区总部。

　　建设基础设施完善、生活配套服务齐全的“工业城”。加快市高新区、银洲湖开发区、江沙工业走廊、广海湾开发区及各市（区）核心工业园区建设，吸引民资外资参与“工业城”开发建设，突出产业特色，形成品牌化、规模化、集约化，提高招商引资载体的建设水平。

　　深化国有企业改革。坚持分类指导原则，推进国有企业投资和产权主体多元化改革，成熟一户，改制一户。加快劣势国有企业退出竞争领域，加大国有资本向社会公共设施投入的力度。强化国有资产监督管理，完善国有资产经营考核和激励分配机制，建立权责统一、有效制衡的企业法人治理结构，探索建立国有资本经营预算制度，提高国有资产营运效益。

　　提高对外贸易水平。促进出口商品结构优化和加工贸易转型升级，提高机电产品和高新技术产品的出口比重。在巩固港澳台、欧美日韩等传统市场的同时，积极开拓非洲、南美、东南亚、中东等新兴市场。加强口岸和“大通关”建设，提高通关效率。进一步完善出口管理部门与出口企业的信息服务平台建设，发挥行业商会（协会）作用，加快建立应对国际贸易摩擦的有效机制，保持外贸出口稳定增长。

　　四、着力建设社会主义新农村，统筹区域和城乡发展

　　强化对农业农村的扶持政策。加大对农业农村的投入力度，今年全市各级财政预算用于“三农”的支出为４５５６８万元，比上年增加８０４１万元，增长２１．４３％；占预算支出的比重达８．２３％。建立和完善以工促农、以城带乡的多元化投入机制，提高支农资金使用效益。大力推进鼓励边远困难地区在投资旺地发展工业的异地发展模式，合理制定和落实税收分成办法，充分调动边远地区招商引资的积极性，促进区域协调发展。今年地方财政收入超千万元的镇（街）再增加３－５个，集体年收入３万元以下的贫困村再脱贫５０个以上。

　　加快中心镇和专业镇建设，推进“三化”进程。全面完成中心镇的控制性详细规划编制工作，加大对中心镇和专业镇的政策倾斜和扶持力度，加快镇域公共基础设施建设，统筹规划建设区域性的供水、污水处理和垃圾处理系统，新建１１５公里农村水泥路。加强对农民的职业技能培训，促进农村富余劳动力加快转移。大力培育扶持农业龙头企业和农民专业合作经济组织。继续抓好以市农产品质量监督检验测试中心为龙头的检测体系建设。推进农业标准化工作，建设农业标准化示范区。抓好农田水利基础设施建设，提高农业抗风险能力。加强农业科技推广服务工作，大力发展特色农业、精品农业和外向型农业，增强农产品的市场竞争力。抓好“万村千乡”市场建设试点工作，推进农村市场体系建设，着力解决农村市场建设滞后、产销脱节、农民增产不增收的问题。继续深入开展“十百千万”干部下基层驻农村活动，推动薄弱镇村经济加快发展。

　　加强规划管理，营造农村新风貌。按照“省管到县、市管到镇、县管到村”的要求，理顺镇村规划管理体制，搞好镇村规划建设管理。抓好农村改水、改厕、改路、改灶、改房工作，今年继续改造农村危房１０００户，确保明年提前完成全市农村危房改造任务。大力整治“脏、乱、差”，积极推广农村沼气应用，改善农村生态环境。加强农村卫生院、卫生站建设，健全三级医疗网络，着力解决农村看病难、看病贵问题。抓好社会主义新农村示范村建设，以点带面，扎实推进。

　　深化农村综合改革，提高镇村民主管理水平。推进“并镇、并村、并校”，抓好乡镇综合配套改革试点，化解镇村历史债务，进一步提高乡镇政府社会管理和公共服务水平。强化涉农收费和农资价格监管，健全对被征地农民的合理补偿和安置机制。规范村民议事和重大村务处理程序。强化村民理财和财务监督机制，完善村干部和村级财会人员离任审计制度，加强村级财务管理。建立健全考核激励机制，加强检查评审和执法监督，推动村务公开和民主管理工作有序健康发展。

　　五、着力加强交通基础设施建设，提升城市区位优势

　　加快港口、铁路、轻轨和高速公路等交通网络的规划、衔接与建设，配合落实江珠高速公路、城际轻轨江门支线和广珠铁路江门段建设的相关工作，争取江肇高速公路工程早日开工。加大国、省、县道改造力度，加快国道３２５线鹤山路段大修工程、双和公路、开平大道等项目建设。确保滨江大道明年５月底前建成通车。加快江门市区北新区汽车客运站场、新会区汽车总站和各中心镇客运站等公交站场建设。推进银洲湖港口群和崖门出海航道、劳龙虎水道整治工程建设。发展数字电视和第三代移动通信业务，提升信息化基础设施水平。

　　六、着力发展物流、金融、旅游业，提高现代服务业水平

　　大力发展现代物流业。制定全市物流业发展规划，引进实力强、服务优的现代物流企业，提升我市在泛珠三角物流链中的地位和作用。抓好新会港、江会商贸走廊、江门港和台山广海港等物流基地建设，做好银洲湖保税物流中心申报工作，推动第三方物流业务的开展。积极培育专业市场，重点抓好新会不锈钢、鹤山鞋业鞋材、开平水暖卫浴和纺织服装、恩平麦克风等专业市场的规划建设。发展社区连锁经营，鼓励大中型流通企业到乡村设立连锁店、各类中小型企业自愿结合建立连锁店，推动连锁经营向服务业的广度和深度扩展。积极引导企业、各类资本参与农村商品流通网络建设，进一步改善农村消费购物环境，促进农民消费。

　　构建金融服务新平台。加强银地、银企合作，畅通政府、银行、企业之间密切沟通的渠道，积极引进境内外金融机构到我市开展业务。协助金融机构在我市举办融资推介会、授信推介会，努力优化民营企业、中小企业的融资环境。鼓励发展中小企业信用担保机构和信用咨询机构，支持和引导信用担保机构增资扩股。推进社会信用体系建设，营造金融发展所必需的诚信氛围。

　　提高旅游业发展水平。加大“中国第一侨乡”、“中国优秀旅游城市”的宣传推介力度，做好旅游整体形象包装，打响碉楼与村落、温泉、海岛等旅游品牌。精心办好第二届华人嘉年华暨２００６中国（江门）侨乡旅游节。以线路整合为重点，丰富旅游景点体系，加快冯如航园、赤水温泉等新项目建设，开发银湖湾滨海湿地公园、台山大襟岛中华白海豚保护区、北陡奇石等具有较高旅游开发价值和开发潜力的项目。健全与港澳及泛珠三角地区主要旅游城市之间的旅游合作联络机制，发展紧密协作关系。进一步挖掘旅游项目的文化内涵，提高档次和水平。加强旅游市场管理，规范市场秩序，提高服务质量。

　　七、着力加强绿色江门建设，推动经济、社会与环境的可持续发展

　　大力发展循环经济。以建设节约型城市为目标，推进清洁生产、资源节约和综合利用工作，构建企业、区域和社会三个层面的循环经济框架，促进双水电厂、国洋电厂热电联供，抓好新会双水拆船钢铁有限公司、甘化股份有限公司国家级循环经济试点建设，推动银洲湖纸业基地建成循环经济示范基地。加快建筑墙体材料革新，推动新建住宅和公共建筑节能。

　　提高环境综合整治水平。突出抓好水环境综合整治，加强饮用水源保护，加大重点河段、城镇内河涌和江门河流域的综合整治力度。加快污水处理厂和截污管网建设，确保台山、开平、鹤山（二期）和恩平的城市污水处理厂在年内建成投产，新会区双水镇、台山市广海镇的生活污水处理厂要加快管网建设并在年内投入使用，开平市水口镇、鹤山市共和镇、蓬江区荷塘镇的生活污水处理厂在年内建成；加紧文昌沙水质净化厂扩建工程、新会区东郊污水处理厂二期工程、江海污水处理厂及蓬江区棠下镇污水处理厂建设。

　　加大环保监管力度。推进环保长效监管机制建设，对重点流域、重点行业和重点企业实行挂牌督办、限期整治、动态管理，对纳入省市重点监控和整治的重点污染源及全市水泥生产企业，实行跟踪督查，督促企业按期完成整治任务。创新“潭江联合保护模式”，加快重点水污染企业、污水处理厂的在线监测和远程监控设施建设，实时掌握污染源排污情况，严厉打击偷排漏排行为。对重点行业设立“环保投诉奖”，建立环保监督员热线联系制度，对群众投诉的环保热点问题开展专项整治行动。积极探索工业污染治理市场化机制。严格执行建设项目环境影响评价和环保“三同时”制度，严把招商引资绿色门槛关。

　　加强生态保护。完成《江门市生态市建设规划》编制工作，开展生态示范村、全国环境优美乡镇、全国绿化模范城市创建工作。抓好江门红树林和恩平七星坑两个省级自然保护区的申报和建设工作，继续抓好鹤山大雁山省级森林公园的申报和江门河排国家级森林公园的创建工作。强化森林防火目标管理，落实以镇、村为重点的森林防火责任制，提高预防和扑救森林火灾综合能力。

　　八、着力加快文化名市建设，促进社会各项事业全面发展

　　提高公民现代文明素质。以创建国家文明城市为抓手，深入开展以“爱国、守法、诚信、知礼”为主题的现代公民教育活动，积极弘扬“开拓创新、文明开放、勤劳创业、团结奉献”的侨乡精神。加强大学生思想政治教育和未成年人思想道德建设。抓好市青少年宫建设。繁荣发展哲学社会科学，倡导社会公德、职业道德和家庭美德等社会基本道德规范，倡导科学精神和文明健康的生活方式。

　　加快推进科教兴市战略。加大教育投入，加快教育强市（区）、强镇建设，推进中小学规范化建设，巩固和提高九年义务教育。突出抓好农村基础教育，从今年秋季起全面实行农村免费义务教育，免除农村户口学生义务教育阶段杂费。加快普及高中阶段教育，大力发展职业教育和民办教育，实施万名技师培训工程，办好江门市职业教育展示暨蓝领人才交流会。继续促进高等教育，积极支持五邑大学和江门职业技术学院的发展，确保市职业技术学院一期工程如期建成。加强师资队伍建设，提高教育教学质量，坚决查处教育乱收费行为，让市民对教育有较高的满意度。组织实施全民科学素质行动计划，抓好科学教育与培训基础工程、科普资源开发与共享工程、大众传媒科技传播能力建设工程和科普基础设施工程建设，重点提高未成年人、农民、城镇劳动人口和公务员的科学素质，建设全民学习、终身学习的学习型社会。

　　加快文化产业发展。构建公共文化服务体系，大力开展文化下乡、文化进社区活动。把华人嘉年华活动打造成侨乡文化品牌。办好五邑华侨华人博物馆和江门美术馆馆展。加强对侨乡文化的研究与宣传，推进对外文化交流与合作。全力做好开平碉楼与村落申报世界文化遗产的冲刺工作。建立健全文化产业多渠道的投融资机制，推动以动漫为主体的数字娱乐等新兴文化产业发展，做大文化产业规模。

　　发展卫生、体育等社会事业。努力建设中医药名市；加大公共卫生经费投入，完善公共卫生体系，发展城乡社区卫生服务网络，建设公共卫生重点工程，严密防控重大传染病。建设社区文化体育广场，进一步开展群众性体育运动；打造“国家网球训练基地”品牌；重振台山“排球之乡”雄风；积极备战省运会。认真贯彻新修订的妇女权益保障法，切实保障妇女、未成年人合法权益。完善人口与计划生育目标管理责任制，稳定低生育水平，提高出生人口素质，综合治理出生人口性别比偏高问题，人口自然增长率控制在５．６‰以内。巩固双拥优抚安置工作成果，争创“全国双拥模范城”四连冠。做好新形势下侨务、外事、港澳台事务工作，推进新闻、出版、人防、气象、宗教、老龄、档案、地志等工作。

　　九、着力解决人民最关心、最直接、最现实的利益问题，努力构建和谐江门

　　优化创业就业环境。把扩大就业摆在经济社会发展的突出位置，新增就业岗位２万个，城镇登记失业率控制在３．５％以下。建立城乡并重的就业制度和规范有序的劳动力市场，为农民到城镇务工经商营造公平环境。加强职业培训和技工教育，重点提高农村劳动力素质，让每个被征地农民都能免费接受就业培训。完善就业服务体系，落实再就业政策，健全对困难群众的就业援助制度，鼓励劳动者自主创业和自谋职业，促进多形式就业。继续适当提高公务员和教师的收入水平。

　　健全社会保障和救助体系。认真解决群众关心的热点难点问题，加大实施“十项民心工程”的力度。抓好低保工作，做到“应保尽保”。适当提高城乡居民最低生活保障标准，全面落实五保供养政策。以规范社保费征缴为重点，进一步完善社会保障体系。深入推进新型农村合作医疗制度改革，把农村合作医疗筹资标准从人均３０元提高到５０元。完善城乡医疗救助制度，抓好新会区、开平市的医疗救助试点工作。加快解决城镇特困户“住房难”问题，建设城乡安居工程，建立以经济适用住房为主、廉租住房为辅的社会住房保障体系。建立健全红十字会工作网络。发展社会慈善事业。关心残疾人等弱势群体，做好残疾人康复工作。加大殡改工作力度，巩固殡改工作成果。

　　加强社区建设。把城市管理的重心放在社区，理顺社区运作关系，增强社区服务功能，改善社区服务设施，推行社区范围内的单位文体设施资源共享，提高社区建设成效。健全社区调解网络，完善社区维稳机制，建立社区治安防控体系。重拳打击黑恶势力和“两抢两盗”、赌博、制毒贩毒、“六合彩”等违法犯罪活动。加强普法教育，加强流动人口和出租屋管理，抓好监狱、劳教工作，重视做好刑满释放人员帮教和社区矫正工作。创建无毒社区和平安社区，城镇“平安社区”覆盖面达到社区总数的５０％以上，市民安全感进一步增强。

　　以铁的手腕抓好安全生产。建立健全重大危险源登记建档制度、重大安全隐患整改督办制度和重特大事故责任追究制度，严格落实安全生产责任制和安全生产许可制度，健全企业注册安全主任制度。加大以企业为主的安全设施投入，改善安全生产条件。加大安全综合监管和执法力度，重点加强对危险化学品、非煤矿山、消防、交通、建筑施工的安全监管。提高监管水平，将安监专业队伍建设延伸至镇（街）一级。强化对食品、药品、餐饮卫生等生产和市场监管，保证人民群众健康安全。

　　健全有效的矛盾疏导机制。加强劳动保障监察执法，发挥调解、信访、听证、仲裁等机制作用，引导群众按法律程序合理表达利益诉求，从源头上预防和减少群体性事件的发生。

　　十、着力推进行政管理创新，加强政府自身建设

　　提高科学民主决策水平，建设民本型政府。把《江门市可持续发展战略纲要》转化为政府决策。坚持经济形势分析联席会议和经济社会发展顾问咨询制度；深入调查研究，及时掌握社情民意和经济社会发展的新情况、新问题；健全重大决策和重要规划的论证和听证程序，认真听取人大代表、政协委员、各民主党派、工商联、无党派人士的建议和意见，确保人民群众发表意见、反映问题的言路畅通。

　　严格依法行政，建设法治型政府。进一步抓好行政许可法、公务员法的贯彻落实，提高政府全体工作人员的法律意识和依法行政能力，规范各类行政执法行为。自觉接受人大及其常委会的监督，切实办好人大代表议案、建议和政协委员提案，支持工会、共青团、妇联、工商联等人民团体发挥作用。推进政务公开和电子政务建设，打造“阳光政府”。完善公共财政体制，加强财政绩效评价，提高财政管理水平。严格依法征税，创新税源管理，依法加强对改变住宅和工业用途的建筑（即住改租、住改商、工改商）的税费征收。严格依法规范政府举债行为，逐步消化政府债务。

　　坚持求真务实，建设服务型政府。服务基层要到位，凡由市直部门审批、核准和备案，或需上报省办理的，各市、区都可以通过市行政服务总汇全程代办，杜绝拖拉、推诿现象。服务市民要到位，进一步扩大“１２３４５”政府热线服务覆盖面，优化服务水平，全力解决市民反映的热点难点问题。

　　提高队伍素质，建设效率型政府。以在市直机关全面导入ＩＳＯ质量管理体系为着力点，推进管理创新和制度创新，加强公务员队伍思想教育，加大公务员培训工作力度，提高公共管理服务的能力和水平。提倡开短会、说短话，切实改进工作作风。

　　自觉接受监督，建设廉洁型政府。健全预防和惩治腐败体系，坚持不懈地开展反腐败工作，落实廉政责任制。坚决整治损害群众利益的行业不正之风，对群众投诉多、意见大的政府工作人员，应先调离原工作岗位；坚决依法清除公务员队伍中的“害群之马”。严格执行建设工程项目招投标、经营性土地使用权出让、产权交易和政府采购等制度，从源头上防止腐败。

　　各位代表，今年是实施“十一五”规划的头一年，是江门侨乡实现跨越式发展的重要一年，我们肩负的历史使命光荣而艰巨。我们要在中共江门市委的领导下，团结全市人民，齐心协力，把握机遇，扎实工作，奋发有为，为实现“十一五”规划宏伟目标，建设富裕、绿色、和谐江门而努力奋斗！
